# Habitat foraging niche of a High Arctic zooplanktivorous seabird in a changing environment

**DOI:** 10.1038/s41598-017-16589-7

**Published:** 2017-11-24

**Authors:** Dariusz Jakubas, Katarzyna Wojczulanis-Jakubas, Lech M. Iliszko, Hallvard Strøm, Lech Stempniewicz

**Affiliations:** 1 Department of Vertebrate Ecology and Zoology, Faculty of Biology, University of Gdańsk, Wita Stwosza 59, 80-308 Gdańsk, Poland; 2 Norwegian Polar Institute, Fram Centre, Postboks 6606, Langnes, 9296 Tromsø, Norway

## Abstract

Here, we model current and future distribution of a foraging Arctic endemic species, the little auk (*Alle alle*), a small zooplanktivorous Arctic seabird. We characterized environmental conditions [sea depth, sea surface temperature (SST), marginal sea ice zone (MIZ)] at foraging positions of GPS-tracked individuals from three breeding colonies in Svalbard: one located at the southern rim of the Arctic zone (hereafter ‘boreo-Arctic’) and two in the high-Arctic zone on Spitsbergen (‘high-Arctic’). The birds from one ‘high-Arctic’ colony, influenced by cold Arctic water, foraged in the shallow shelf zone near the colony. The birds from remaining colonies foraged in a wider range of depths, in a higher SST zone (‘boreo-Arctic’) or in the productive but distant MIZ (second ‘high-Arctic’ colony). Given this flexible foraging behaviour, little auks may be temporarily resilient to moderate climate changes. However, our fuzzy logic models of future distribution under scenarios of 1 °C and 2 °C SST increase predict losses of suitable foraging habitat for the majority of little auk colonies studied. Over longer time scales negative consequences of global warming are inevitable. The actual response of little auks to future environmental conditions will depend on the range of their plasticity and pace of ecosystem changes.

## Introduction

Climate change affects biological systems across the globe. The Arctic is warming faster than other regions of the Earth^1^, the process likely amplified by several positive feedbacks that increase temperature at a rate of 2–3 times higher than the global average^1,2^. The atmospheric warming and increase in the Arctic Ocean temperature have already resulted in decreased extent and thickness of sea ice, with the sea ice extent currently decreasing by 10% per decade^1,3^. Increase in temperature in Arctic seas as well as shifts in water masses and hydrological fronts may have serious consequences including changes in species distributions and the whole marine ecological communities^2,4^.

Endemic Arctic species, using sea ice habitat [e.g. sympagic amphipods, polar bears (*Ursus maritimus*) or narwhals (*Monodon monoceros*)], are arguably species most vulnerable to the consequences of climate change^2^. Considering the pace of changes, organisms may only adapt or respond through microevolution or phenotypic plasticity, or by migrating to suitable habitats^4,5^. Limited literature exists documenting the impacts of current climate change in Arctic on marine biota based on long-term data (e.g. refs^6–8^). With limited reliable baseline information, it is important to model current distribution of endemic Arctic species to recognize important features that shape their habitat niche^2^. This knowledge is crucial in the preparation of realistic scenarios of responses to climate change that, in turn, may help to assess whether distribution ranges of the species will shift, expand or contract.

The little auk, or dovekie (*Alle alle*) is an endemic Arctic organism. It is a small zoopolanktivorous seabird whose breeding range is restricted to the High Arctic^9^. Its foraging is energetically expensive^10,11^ due to high costs of locomotion in the air (flapping flight). The little auk forages on Arctic copepods, that are larger and much richer in energy than their counterparts from warmer Atlantic waters (e.g.^12–14^). Female little auks lay a single egg which is incubated by both parents. The chick is brooded for the first few days and fed by both parents^9^. Considering the high energetic requirements, diet preferences and distribution, the little auk is an excellent model species for forecasting the effects of future climate change in Arctic. Due to its abundance (>37 million pairs^15^), the little auk is an important component of High Arctic ecosystems and transports huge amounts of organic matter from sea to land, fertilizing the nutrient-deprived Arctic tundra^16–18^.

Here, using remote sensing data, tracks of GPS-logger-equipped little auks and modelling approach, we characterize the current foraging environment (sea surface temperature, sea depth, presence of sea ice, distance from the colony) of the species across Svalbard. Given current feeding habitat characteristics of the species, we further predict future distribution of the birds foraging areas in the context of climate amelioration. We consider two scenarios of increase in sea surface temperature by 1 °C and 2 °C. We expect that current optimal habitats for foraging little auks are mainly limited to shelf waters influenced by cold Arctic-origin water masses and to deeper waters at the marginal sea ice zone. As those optimal habitats may be threatened by climate change, we predict a future reduction of foraging habitats suitable for little auks, especially those in southern Svalbard.

## Methods

### Study area

**Figure 1:**
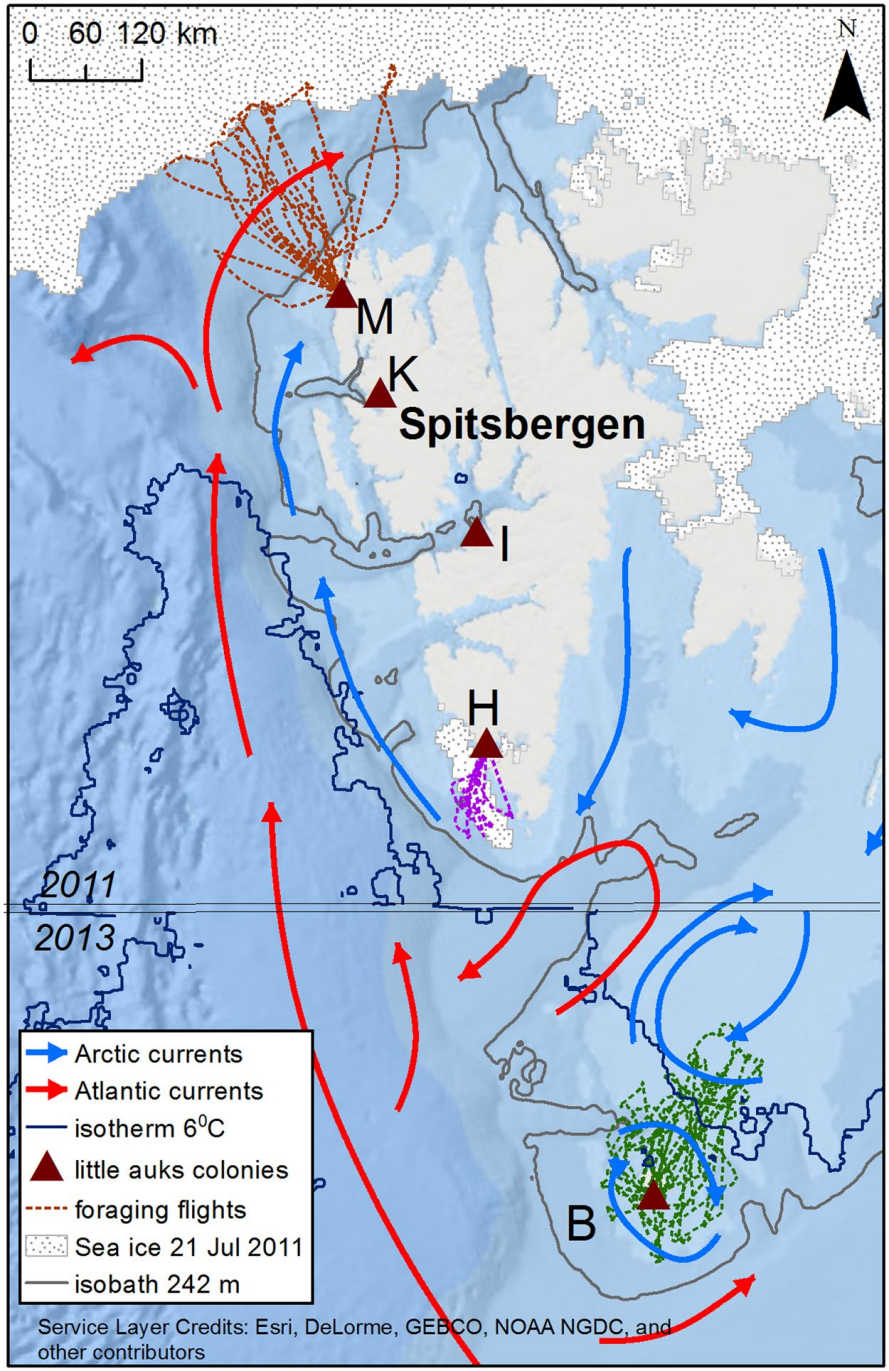
Study area with foraging trips of GPS-logger equipped little auks^36,38^. Ocean currents after^22^, modified. Sea ice extent recorded on 21 July 2011 coinciding with GPS-tracking in Magdalenefjorden and Hornsund (based on ice maps from^46^, with 4 km resolution). Little auk colonies are denoted with capital letters: B – Bjørnøya, H – Hornsund, I – Isfjorden, K – Kongsfjorden, M – Magdalenefjorden. A 242 m isobath reflects the multiyear cut-off point value for Arctic zooplankton occurrence in the Hornsund area^31^. Isotherm 6 °C (right) reflects physiological threshold for *Calanus glacialis* functioning in Svalbard^54^. Horizontal double line with years 2011, 2013 indicates line of two maps fusion in SST mosaic. Map was produced in ArcMap 10.3.1 (Redlands, CA: Environmental Systems Research Institute) using bathymetry map (Arctic Ocean Base, https://services.arcgisonline.com/arcgis/rest/services/Polar/Arctic_Ocean_Base/MapServer) and Multisensor Analyzed Sea Ice Extent **–** Northern Hemisphere (MASIE-NH) data (http://nsidc.org/data/docs/noaa/g02186_masie/).

Svalbard is a High Arctic archipelago in the Arctic Ocean. It comprises a number of islands including the largest, Spitsbergen and the southernmost, Bjørnøya. The archipelago is influenced by cold Arctic water masses from the north-east and the warm West Spitsbergen Current flowing northwards along its western coast (Fig. 1). The latter current is the main source of Atlantic water in the Arctic Ocean^19^ and is the dominant heat source for the Arctic Ocean^20^. In the Svalbard area, Arctic and Atlantic waters meat at the Polar Front, the position of which is strongly influenced by bottom topography^21,22^. Thus, the waters around Svalbard area are mixture of cold, less saline Arctic, and warmer, more saline Atlantic waters forming a confluence zone where the three dominant *Calanus* species co-occur: *C. finmarchicus* (Atlantic) *C. hyperboreus, and C. glacialis* (Arctic)^23^. The Svalbard Archipelago has experienced very rapid air temperature increases in recent decades and this trend is expected to continue unabated through to the end of this century^24^. The summer position of the marginal ice zone around Svalbard has shifted in recent years northward from the continental shelf zone to the deep Arctic Ocean Basin^25^. Currently, most Arctic endemic species in Svalbard are experiencing negative consequences induced by the warming environment^26^.

This study focused on three distinct breeding colonies of the little auk in Svalbard. Two large colonies were situated in Spitsbergen fjords: in Hornsund (SW Spitsbergen; 77°00′N, 15°33′E) and in Magdalenefjorden (NW Spitsbergen; 79°35′N, 11°05′E). Both fjords are considered as the main breeding aggregations of the little auk in Svalbard^27^ with breeding populations estimated at 449,000 pairs in Hornsund and 18,000 pairs in Magdalenefjorden (Keslinka, unpublished data). The third studied colony, on Bjørnøya (74°31′N, 19°01′E), estimated at 10,000 breeding pairs^28^, represents the southernmost Svalbard Little auk population. Also included in analyses of future distribution of foraging areas were two smaller colonies in Central W Spitsbergen, for which no GPS-tracking data exists: Isfjorden (78°12′N, 15°20′E) and Kongsfjorden (79°01′N, 12°25′E), where the populations were estimated at 230 and 3,900 pairs, respectively (Keslinka, unpublished data) (Fig. 1).

The foraging grounds of birds at the studied colonies differed in environmental conditions. Bjørnøya is a small island in the western Barents Sea, near the south-western end of the shallow Spitsbergen Bank that, in summer, is characterized by a mixture of cold Arctic water and melt-water from the Barents Sea ice pack. It is surrounded to the west, south and southeast by warm Atlantic water masses^14^ (Fig. 1). We consider this colony, located at the southern rim of the Arctic zone and thus especially prone to climate-change induced shifts in position of atmospheric and oceanic circulation patterns as ‘boreo-Arctic’. The remaining colonies situated north off Bjørnøya, on Spitsbergen, we consider as ‘high-Arctic’. The Hornsund area is influenced by both the coastal, cold Arctic Sørkapp Current and the warm West Spitsbergen Current (WSC) that transports Atlantic water from the Norwegian Sea^29^. In the Hornsund area, sea ice is present only in some seasons depending on atmospheric and oceanic processes^30,31^. Isfjorden and Kongsfjorden are open fjords (i.e. without sill at the mouth of the fjord) in the central part of the west coast of Spitsbergen. Both fjords and the adjacent shelf-sea area outside the fjords function under a balance of influx of Atlantic waters from the West Spitsbergen Current and Arctic water from the Sørkapp Current^32,33^, with inter-annual variations in the inflow of Atlantic water also common^29^. In the Magdalenefjorden area, the warm West Spitsbergen Current flows along the shelf slope and meets relatively cold and fresh Arctic water that arrives as a coastal current. Both water masses partly mix, creating a transitional zone^34^. The marginal sea ice zone is more or less permanently situated ca. 100 km north of Magdalenefjorden in the summer (Fig. 1).

### Location of foraging areas

To determine little auk foraging areas we used foraging positions of GPS-logger equipped birds recorded during the early chick-rearing period at three colonies over two seasons, Hornsund and Magdalenefjorden (both in 2011) and Bjørnøya (in 2013). We used miniature GPS loggers (Ecotone, Sopot, Poland) weighing 4.2–4.5 g including attachment that is equivalent to 2.3–3.3% of the little auk’s body mass^35–38^. Results on foraging of GPS-logger equipped little auks from colonies in Hornsund, Magdalenefjorden and on Bjørnøya have been already published^36,38^.

All animal research protocols were carried out in accordance with guidelines for the use of animal^39^ and approved by Norwegian Animal Research Authority and the Governor of Svalbard. More details about protocols and effects of loggers on birds are provided in previous studies^36,38^.

We distinguished foraging position based on momentary flight speed (km h^−1^) recorded by GPS-loggers. We assumed values ≤10 km h^−1^ as foraging position as they are considered to be associated with swimming and feeding^40,41^; more details about the loggers deployment and analyses of GPS data are provided in previous papers^36,38^.

### Environmental data

To characterize environmental conditions at the foraging grounds, we used remote-sensed satellite data on sea depth, sea surface temperature (SST), and location of marginal sea ice zone, i.e. factors recognized as important determinants of the occurrence of Arctic zooplankton in Svalbard^31,42^, preferred by little auks during the chick-rearing period on West Spitsbergen^13,43^.

We extracted sea depth data from 500 m global relief model of Earth’s surface IBCAO ver. 3^44^. We obtained 4.63 × 4.63 km resolution data on sea surface temperature (SST) from^45^ provided by the Integrated Climate Data Center, University of Hamburg, Hamburg, Germany (http://icdc.cen.uni-hamburg.de/1/daten/ocean/sst-modis.html). We processed satellite images in SeaDAS 7.3.1 (http://seadas.gsfc.nasa.gov/) software before further analyses in ArcMap 10.3.1 (Redlands, CA: Environmental Systems Research Institute).

**Figure 2:**
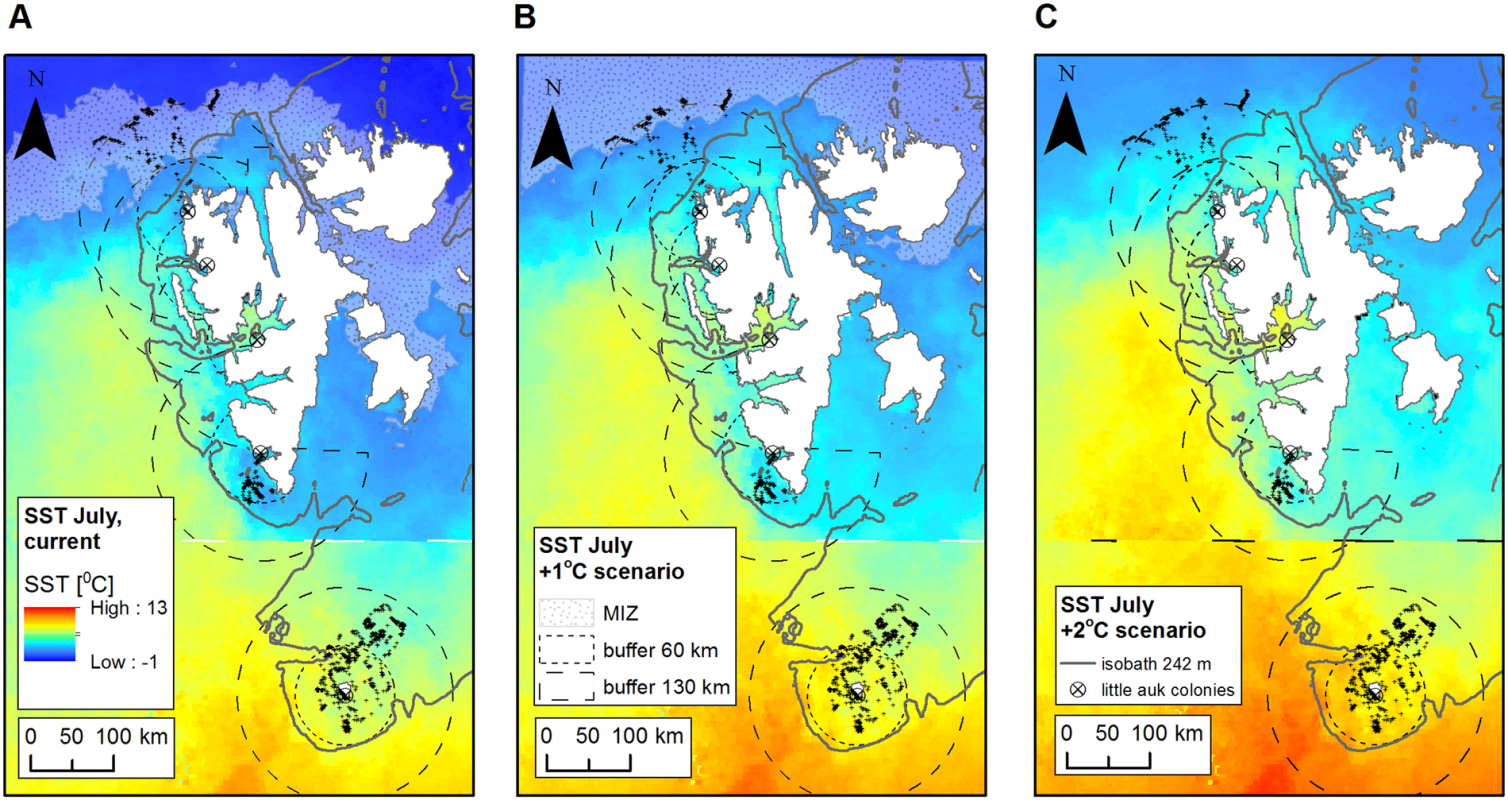
Environmental characteristics of little auk foraging habitats (sea surface temperature, marginal sea ice zone and 242 m isobath) under present conditions (**A**) and under +1 °C (**B**) and +2 °C (**C**) scenarios. MIZ - location of marginal sea ice zone. Maps were produced in ArcMap 10.3.1 (Redlands, CA: Environmental Systems Research Institute) based on data of^45^ available from the Integrated Climate Data Center, University of Hamburg, Hamburg, Germany (http://icdc.cen.uni-hamburg.de/1/daten/ocean/sst-modis.html).

We used SST data for July that represents the early and mid chick-rearing periods when the GPS-tracking data were collected^36,38^. For further spatial analyses, we prepared a mosaic of maps of SST in July 2011 for Spitsbergen and in July 2013 for Bjørnøya (Fig. 2). We prepared all maps in ArcMap 10.3.1.

We defined the marginal sea ice zone (MIZ) as the area with SST within the range 0.0–1.5 °C. Its position has been verified by the real sea ice extent recorded remotely [based on the 4 km resolution daily data^46^ (http://nsidc.org/data/docs/noaa/g02186_masie/)] during the flights of GPS-tracked little auks from Magdalenefjorden (Fig. 1). We considered MIZ position in July.

### Analyses - current environmental conditions

We used a Conditional Inference Tree (CIT) to model importance of environmental variables (SST in July, presence of MIZ, sea depth and distance from the colony) at the foraging positions of GPS-logger-equipped little auks from ‘boreo-Arctic’ and two ‘high-Arctic’ colonies. CIT is a non-parametric class of regression tree, robust to typical regression problems such as over-fitting, collinearity, and bias with regard to the types of explanatory variables used^47^. We conducted CIT analyses in R software^48^ using the *party* package^47^. We checked the significance of nodes in CIT analyses using structural change test implemented in the *strucchange* package in R^49^.

### Analyses - fuzzy logic based habitat modelling

We modelled the suitability of the areas around Svalbard (extent of analyses - see Fig. 1) for little auk foraging using a weighted linear combination fuzzy logic model. We used fuzzy linear transformation function for SST in July, fuzzy small transformation function for sea depth and distance from the colony to the foraging grounds and binary function for presence/absence of marginal sea ice zone in July (Table 1). We weighted the particular factors based on expert knowledge giving the highest scores to factors important for *C. glacialis*, i.e. SST and presence of marginal sea ice zone^42^ (Table 1). At the defuzzification stage of analyses, we chose the following two thresholds to predict the distribution of little auk foraging positions:

1. 0.9 - with high accuracy for ‘high-Arctic’ Spitsbergen colonies (97%) but worse performance for all colonies (64%) and ‘boreo-Arctic’ colony (41%). This represents a conservative response to climate change with foraging restricted only to optimal, cold water areas reflecting current feeding habitats of population breeding on Spitsbergen (hereafter ‘restricted response’).
2. 0.7 - with high accuracy for all colonies (92%, in that 87% for ‘boreo-Arctic’ foraging areas around Bjørnøya). This reflects Svalbard-wide plasticity in little auk reaction to environmental changes meaning foraging in a wider range of temperatures reflecting a full range of current feeding niches including both ‘high-Arctic’ and boreo-Arctic’ conditions (hereafter ‘flexible response’).

**Table 1:** Fuzzy weighted linear combination model description.

	Factors			
	SST	Bathymetry	Marginal sea ice zone	Cost distance
Factor description	Sea surface temperature Mosaic raster for July	Sea depth	Temperatures 0.0–1.5 °C on SST mosaic	Euclidean distance from the colonies with high costs of flight over land
Membership function/data type	Fuzzy linear	Fuzzy small	Presence /absence	Fuzzy small
Parameters	Min: 8 °C Max: 6 °C	Midpoint:242 m Spread: 5	—	Midpoint: 219 km Spread: 5
Source of expert knowledge	42	31	35, 36	86
Weight of the factor	0.4	0.2	0.3	0.1

Final score is the sum of all ratings multiplied by the weight of particular factors.

Detailed description of fuzzy logic approach can be found as Supplementary Information. We performed all fuzzy logic modelling in ArcMap 10.3.1.

### Analyses - climate change scenarios

To predict habitat suitability in conditions of predicted climate amelioration, we considered two scenarios – an increase of SST temperature in July by 1 °C and one by 2 °C. A temperature increase of 2 °C by the end of the century is expected in the Barents Sea^50^, and is also considered in scenarios for zooplankton distribution in this area^42,51^. We prepared SST +1 °C and +2 °C scenarios maps in ArcMap 10.3.1 based on maps used in current condition analyses. We also predicted position of MIZ in July by adding 1 °C and by 2 °C to the current values. We implemented those data in fuzzy logic sets describing future conditions (Fig. 2).

To assess current and future conditions on foraging grounds at cost-effective distances from the colony, we calculated area of suitable habitats in 60 and 130 km buffer zones around studied colonies representing medians of maximal straight-line distance to little auk foraging positions recorded in Hornsund (60 km) and Magdalenefjorden (129 km)^36^ and the maximal flight range of individuals from Bjørnøya (132 km)^38^. We excluded land and areas on the east coast of Spitsbergen from the zones. Results of GPS-tracking and at-sea surveys indicate that little auks do not fly over land, and that birds from colonies from the west coast of Spitsbergen do not explore the east coast of Spitsbergen^12,36,52^. We calculated the area of suitable foraging grounds in both buffer zones using present and predicted conditions considering two defuzzification thresholds 0.7 and 0.9 representing flexible and restricted type responses characterized above.

### Data availability

The datasets generated during and/or analysed during the current study are available from the corresponding author upon reasonable request.

## Results

### Current environmental conditions

**Figure 3:**
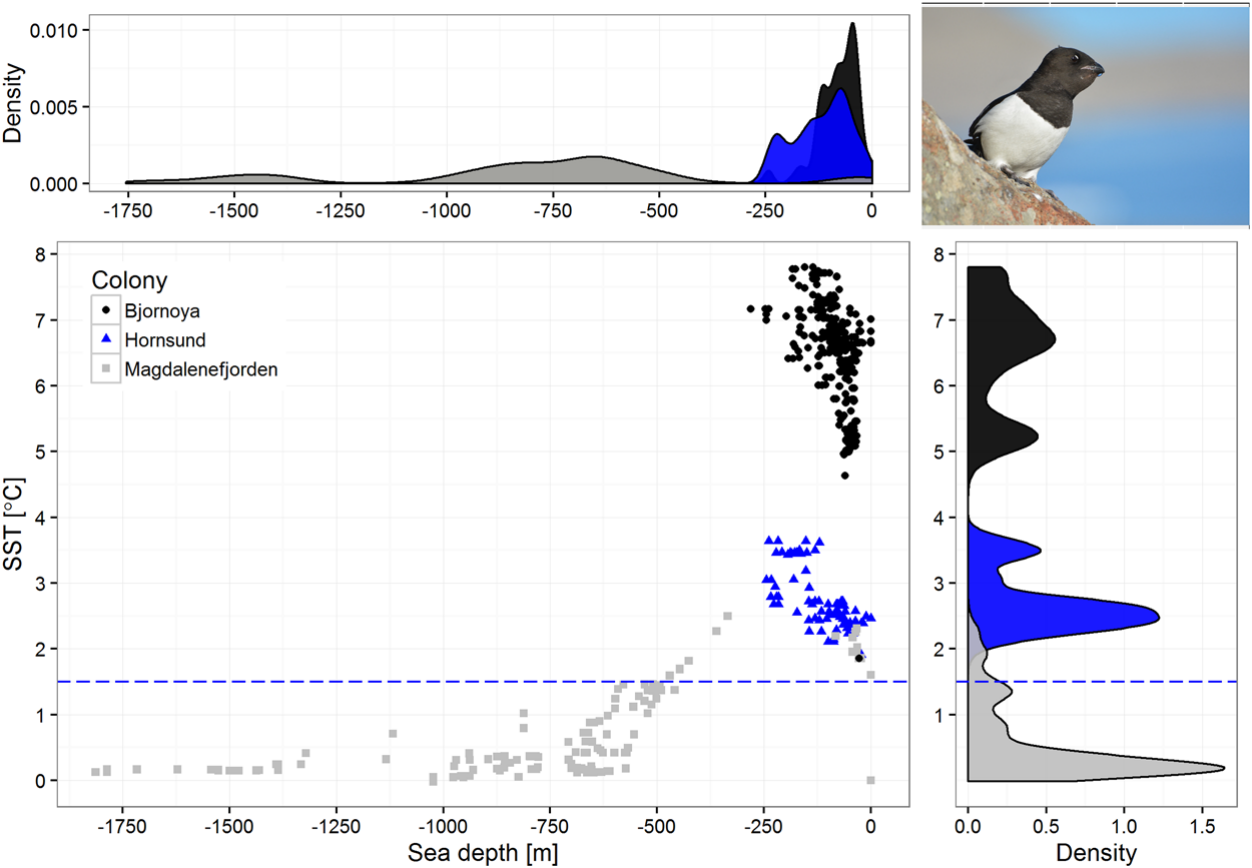
Distribution of sea surface temperature (SST) and sea depth at foraging positions of GPS-logger-equipped little auks breeding on Bjørnøya, in Hornsund and Magdalenefjorden. Density – probability density. Blue dashed lines indicate upper range of SST characterizing marginal sea ice zone. Plot was created in R software version 3.3.2^48^. Photo by Katarzyna Wojczulanis-Jakubas.

**Figure 4:**
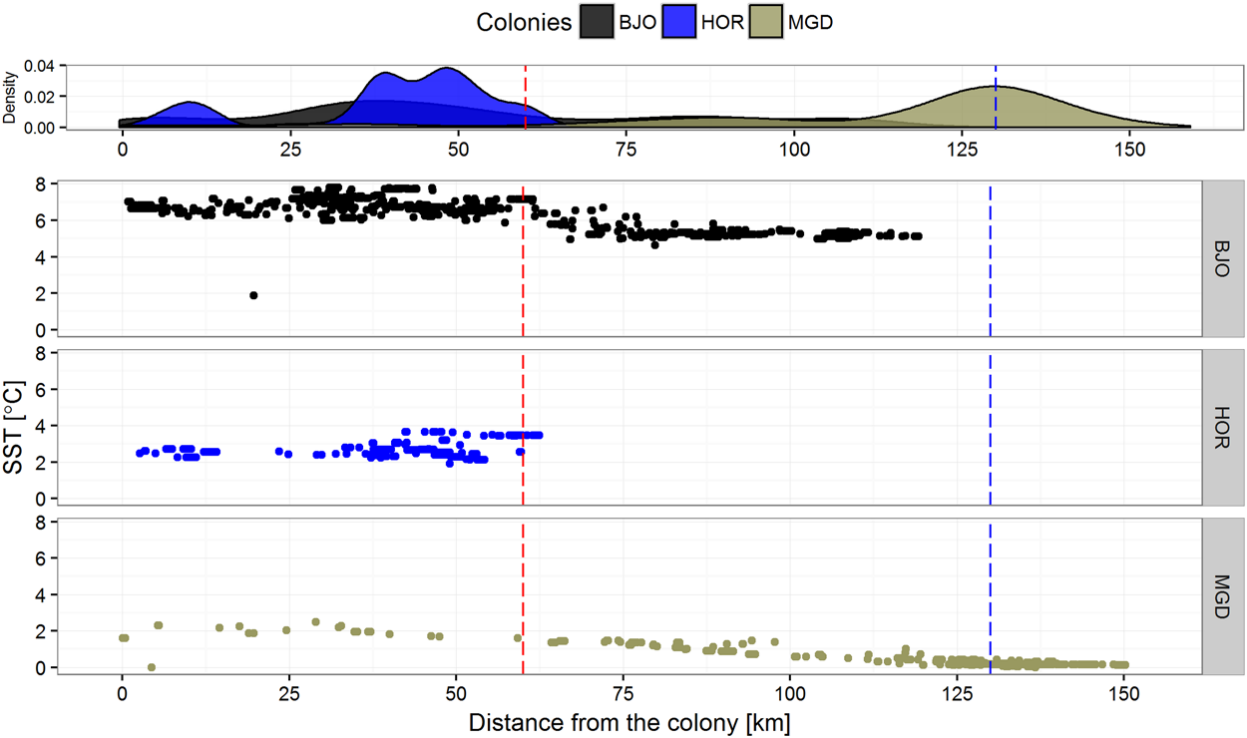
Sea surface temperature (SST) and straight-line distance from the colony to foraging positions of GPS-logger-equipped little auks breeding on Bjørnøya (BJO), in Hornsund (HOR) and Magdalenefjorden (MGD). Red and blue dashed lines indicate travel distance of 60 km and 130 km, representing maximal straight-line distance to little auk foraging positions (Hornsund and Magdalenefjorden) and the maximal flight range (Bjørnøya). Density – probability density. Plot was created in R software version 3.3.2^48^.

The foraging conditions around ‘boreo-Arctic’ Bjørnøya were best characterized by enhanced SST values, up to 7.8 °C (Figs 2, 3 and 4), with no overlap in SST values with the other two, ‘high-Arctic’ colonies (Figs 3 and 4). Birds from ‘high-Arctic’ Hornsund and Magdalenefjorden foraged in significantly colder water (≤3.64 °C) compared to ‘boreo-Arctic’ Bjørnøya. Foraging areas with the lowest range of SST (~0 °C) located over deep water zones (>−500 m) in MIZ were used exclusively by birds from Magdalenefjorden (Figs 3 and 4). Distribution of distances from colony to foraging points differed between all colonies (Fig. 4). Birds from ‘boreo-Arctic’ Bjørnøya and ‘high-Arctic’ Magdalenefjorden foraged both in close (up to 60 km) and distant locations (above 100 km). Distant locations were characterized by a lower SST than closer locations (0–1 °C vs 1.5–2.5 °C in Magdalenefjorden and 4–6 °C vs 6–8 °C on Bjørnøya). In the Hornsund area, little auks foraged in close locations with intermediate SST (2–4 °C) compared to foraging grounds in Bjørnøya and Magdalenefjorden (Fig. 4).

**Figure 5:**
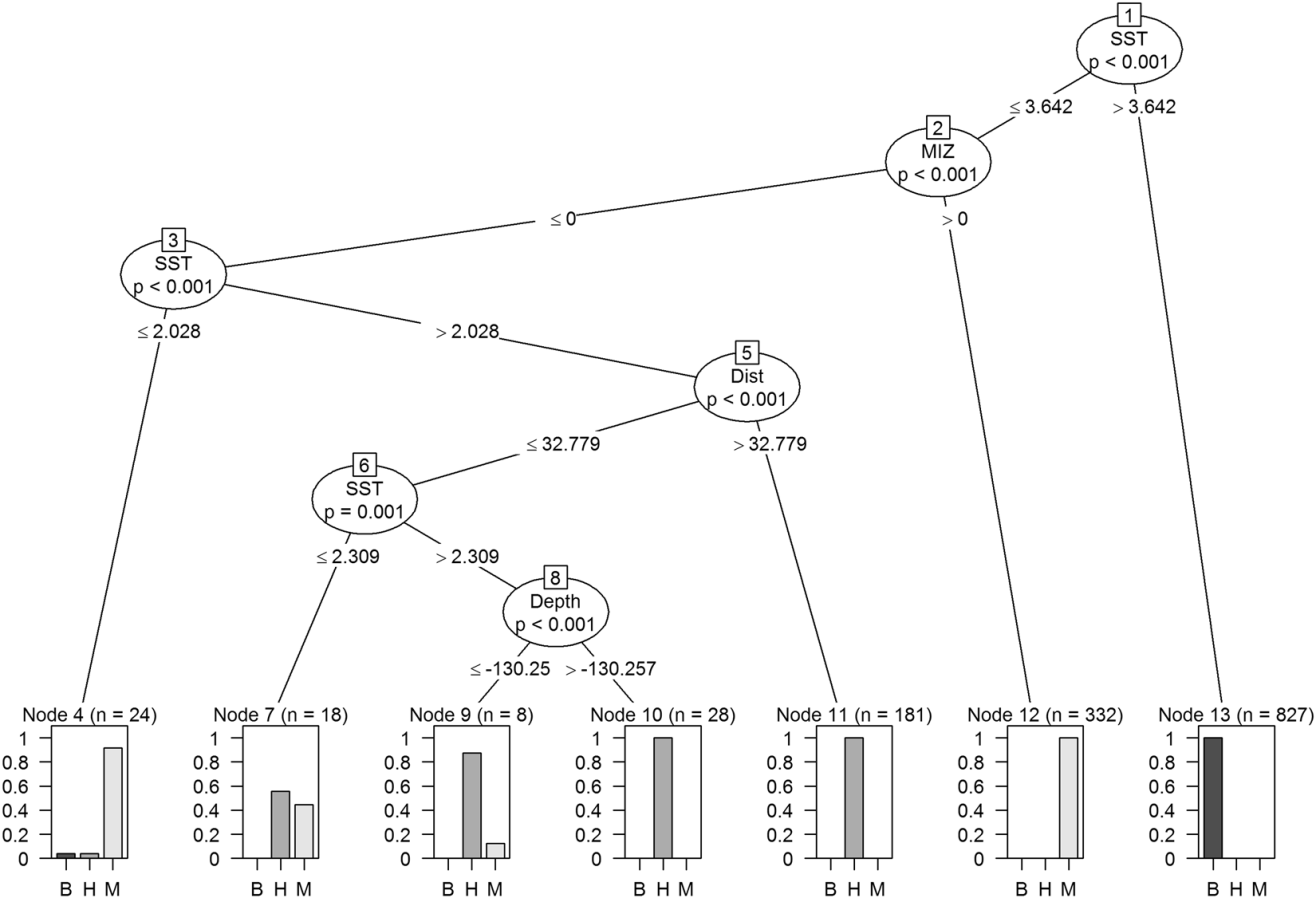
Conditional Inference Tree characterizing environmental conditions in foraging areas of GPS-logger-equipped little auks from colonies on Bjørnøya (B, *n* = 828 points), in Hornsund (H, *n* = 227 points), and Magdalenefjorden (M, *n* = 363 points). The following characteristics of recorded in little auk foraging positions: sea surface temperature in July [°C] (SST), sea depth [m] (Depth), distance to the colony [km] (Dist) and presence/absence of marginal sea ice zone (MIZ) were used as initial explanatory variables. Encircled variables have the strongest association to the response variable (Colony) and best characterizes inter-colony differences. The *p* values listed at each encircled node represent the test of independence between the listed variable (SST, Depth, Dist, MIZ) and the response variable (Colony). Terminal nodes indicate which variable levels little auks foraged within and *n* indicates the number of foraging positions from each colony corresponding to specific SST or bathymetry levels. *Histograms* depict probability of particular values in particular colonies. Plot was created in R software version 3.3.2^48^.

The conditional regression tree show that all input variables, i.e. SST, sea depth, presence of MIZ and distance to the colony, characterized significantly foraging habitat of GPS-equipped little auks (Fig. 5). All foraging points have been split into those with SST values ≤3.6 °C and >3.6 °C (split 1). The higher SST group (Node 11) was represented by positions recorded exclusively around ‘boreo-Arctic’ Bjørnøya. The colder SST group (≤3.6 °C), observed mainly in Hornsund and Magdalenefjorden, has been split further (split 2) given presence (Node 12) and absence of MIZ (Node 3). Node 12 represents exclusively little auks from Magdalenefjorden exploring MIZ. Node 3 representing birds foraging outside MIZ and has been further split by mean SST ≤2.0 °C (Node 4) and >2.0 °C (Node 5). Node 4 is represented mainly by foraging position in Magdalenefjorden and singular positions from Hornsund and Bjørnøya. The group >2.0 °C (Node 5) has been split by distance from the colony into close located ≤32.8 km (Node 6) and distant, >32.8 km (Node 11). The distant foraging positions group is represented exclusively by foraging grounds around Hornsund. Close foraging areas group (Node 6) has been split into two nodes differing in SST, i.e. between 2.0 °C and 2.3 °C and (Node 7) and between 2.3 °C and 3.6 °C (Node 8). The former node is represented by birds from Hornsund and Magdalenefjorden. The latter has been distinguished into two nodes differing in sea depth, i.e. ≤−130.2 m (Node 9) and >−130.2 m (Node 10), respectively. Both nodes 9 and 10 are represented mainly by birds from Hornsund (Fig. 5).

### Habitat modelling of current conditions

**Figure 6:**
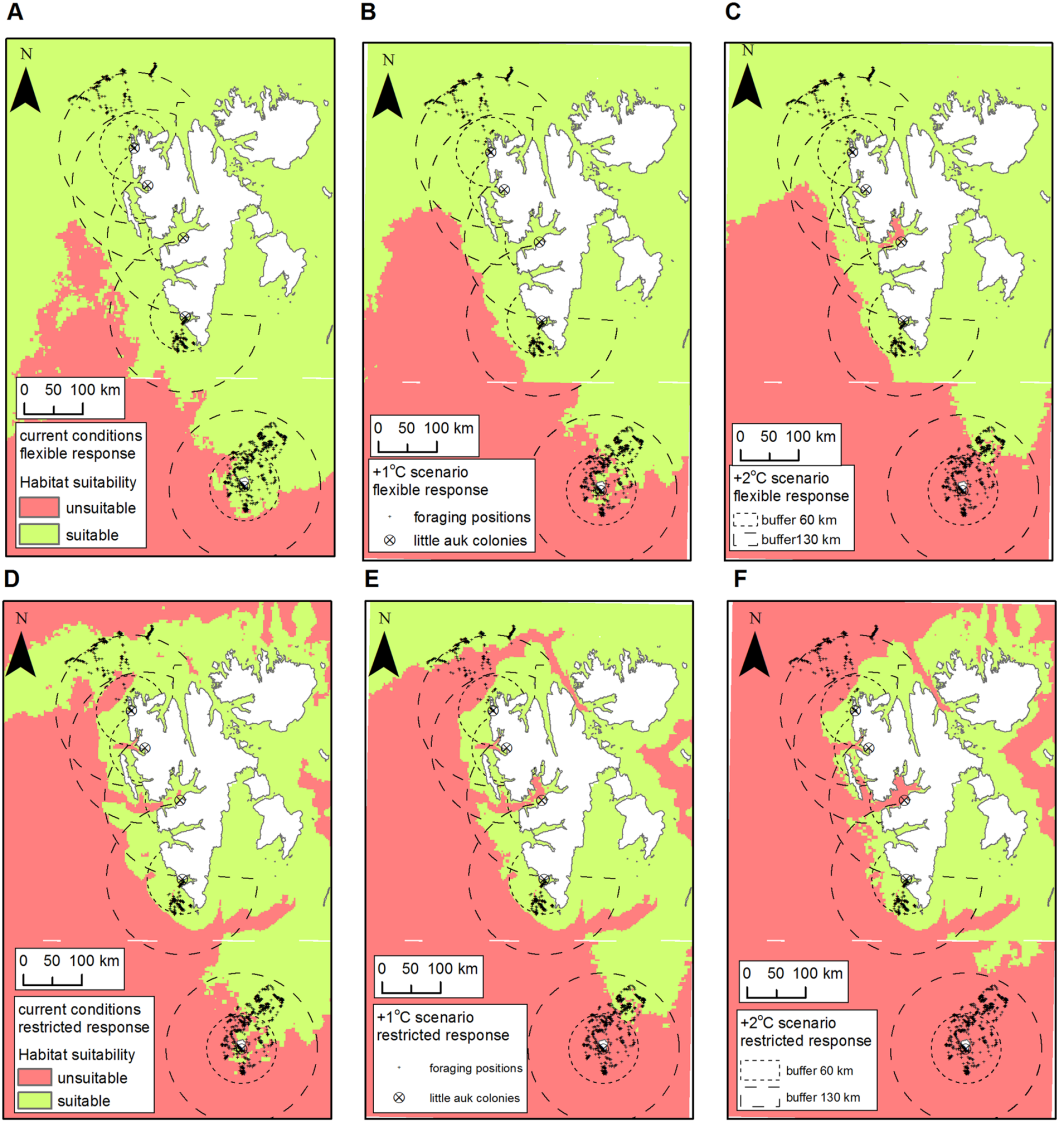
Fuzzy weighted habitat suitability model for the little auks breeding in Svalbard according to flexible response type today (**A**), according to +1 °C (**B**) and +2 °C (**C**) scenarios, and restricted response type, today (**D**), according to +1 °C (**E**) and +2 °C (**F**) scenarios. Flexible and restricted type response reflect two defuzzification thresholds (0.7 and 0.9). Maps were produced in ArcMap 10.3.1 (Redlands, CA: Environmental Systems Research Institute).

**Figure 7:**
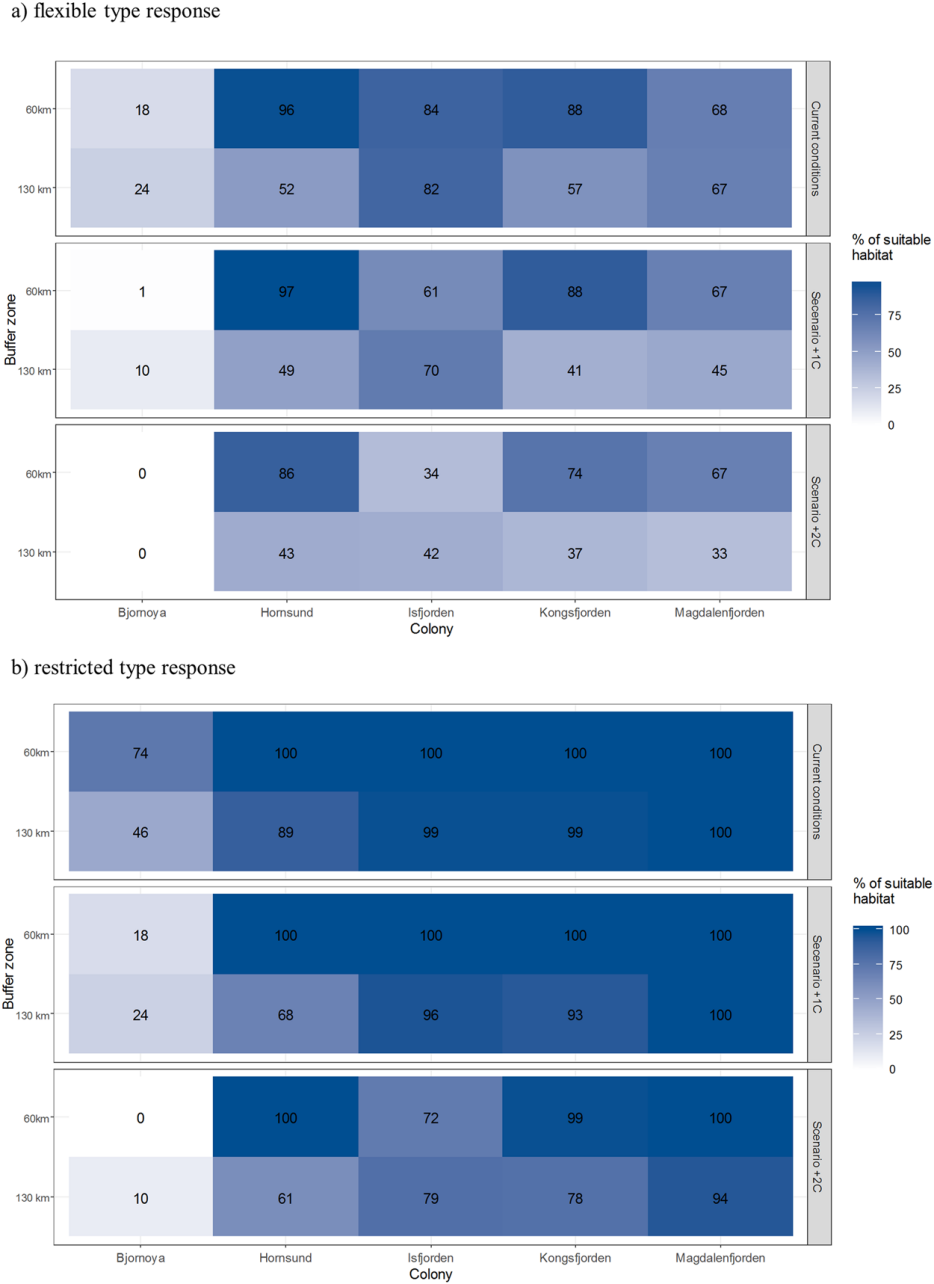
Relative area [%] covered by habitat suitable for foraging little auks (according to fuzzy logic models) within 60 and 130 km buffer zones around the studied colonies under current and predicted future conditions (+1 °C and +2 °C scenarios) according to two defuzzification thresholds (**a**) 0.7 – flexible type response, and (**b**) 0.9 – restricted type response). Plot was created in R software version 3.3.2^48^.

Results of habitat modelling differed between defuzzification thresholds (Figs 6 and 7). According to the restricted type response within the 60 km buffer zone, the largest suitable foraging area was predicted for Hornsund (96%) and the smallest for Bjørnøya (18%). The values for the remaining colonies ranged from 68% to 88%. Within the 130 km buffer zone, the largest area of suitable habitat was predicted for Isfjorden (82%) and the smallest around Bjørnøya (24%). The values for the remaining colonies ranged from 52% to 67%.

According to the flexible type response threshold, all colonies within both buffer zones were characterized by large areas of suitable foraging habitat (>88%), with Bjørnøya providing the exception (46% and 74% in 130 km and 60 km buffer zone, respectively) (Figs 6 and 7).

### Climate change scenarios

Results of habitat predictive modelling differed between scenarios and defuzzification thresholds (Figs 6 and 7). Under the +1 °C scenario and according to the restricted type response within the 130 km buffer zone, little auks from all colonies will experience a loss of suitable habitats compared to the current conditions ranging from −7% in Hornsund to −59% on Bjørnøya. In the 60 km buffer zone, Bjørnøya will experience considerable habitat loss (>99%), Isfjorden will experience moderate loss (−26%) and there will be no considerable loss in the remaining colonies. According to the flexible type response under the +1 °C scenario within both buffer zones, the majority of colonies will be characterized by an area of suitable foraging habitat that is similar to today’s (i.e. loss from 0% to −17%), except for a moderate loss within the 130 km buffer zone in the Hornsund area (−23%) and a considerable loss around Bjørnøya (−48% and −75% in 130 km and 60 km buffer zones, respectively) (Figs 6 and 7).

Under the +2 °C scenario and according to the restricted type response within 130 km buffer zone, little auks from all colonies will experience considerable loss of suitable habitat compared to today, ranging from −18% for Hornsund to a total loss on Bjørnøya. Within the 60 km buffer zone total habitat loss is expected for Bjørnøya, a slight loss for Hornsund and Magdalenefjorden (−10% and −1%, respectively) and a moderate loss for the remaining colonies (−59% in Isfjorden and −15% in Kongsfjorden) (Figs 6 and 7). According to the flexible type response under the +2 °C scenario, Bjørnøya will experience a total loss of foraging habitat within the 60 km buffer zone and a massive loss (−79%) within the 130 km buffer zone. In contrast, some colonies (Magdalenefjorden in both buffer zones, Hornsund and Kongsfjorden within 60 km buffer) will experience only slight habitat losses at the most. We expect moderate habitat losses (20% to −31%) within the 60 km buffer zones in Hornsund, Isfjorden and Kongsfjorden (Figs 6 and 7).

## Discussion

We characterized current and predict future little auk foraging environment in Svalbard, using a combination of GPS-tracking, satellite remote sensing and data-driven (conditional inference tree) and expert-knowledge (fuzzy logic) based techniques. To our knowledge, this is the first study providing an assessment of the foraging niche of a zooplanktivorous seabird in Svalbard based on such a data of high resolution and predicting future distribution of the species foraging grounds under +1 °C and +2 °C water temperatures change scenarios.

### Characteristics of current environmental conditions

All input variables chosen to employ in fuzzy logic model based on expert knowledge (SST, presence of MIZ, sea depth and distance to the colony) were recognized as significant factors in the data-driven conditional inference tree analysis.

The SST ranges recorded at the foraging positions of little auks breeding on ‘high-Arctic’ Spitsbergen (min-max: −0.02–3.6 °C) were in concordance with values (<5 °C) considered as optimal for their main prey, *Calanus glacialis* ^31,42,53^. In contrast, birds from ‘boreo-Arctic’ Bjørnøya foraged regularly in warmer surface waters (percentile 5–100%: 4.6–7.8 °C; Fig. 2). The probability of presence in the Barents Sea of *C. glacialis* is the highest (90–95%) at SST <~6 °C and declines rapidly to <50% at temperatures ~7.4 °C^42^. Temperatures of 5.1 °C and ~6 °C w recognized as the upper limit for the occurrence of the Arctic zooplankton community^31^ and as a threshold for a positive balance between ingestion and respiration in *C. glacialis* in Svalbard^54^, respectively. little auks from some ‘high-Arctic’ Spitsbergen colonies (Kongsfjorden, Isfjorden) also notably foraged, at least partially, in suboptimal areas being influenced by warm Atlantic-origin water masses (SST 4–7 °C). In these waters, dominated by boreal copepods (*C. finmarchicus*), Arctic *C. glacialis* likely functions there at the limit of its physiological threshold (at least in the surface sea layer). Despite these suboptimal conditions, *C. glacialis* still constitutes an important component of little auk diet at mentioned ‘boreo-Arctic’ and ‘high-Arctic’ colonies^38,53,55–58^, indicating that the birds need to work harder to obtain the preferred food items.

Many feeding position of birds from ‘high-Arctic’ Magdalenefjorden were located in MIZ^35,36^. This habitat is rich in the prey attractive for little auks, i.e. *C. glacialis* and the sympagic amphipod *Apherusa glacialis* ^42,59,60^. Indeed, in Magdalenefjorden, 5–18% of the food samples collected from adults consisted almost exclusively of *A. glacialis* ^13,43^. That indicates that the little auks can efficiently exploit even distant waters, which increases the birds foraging flexibility.

We also found that water depth affects distribution of foraging little auk. The biomass of *C. glacialis* in the Barents Sea decreases linearly with water depth, indicating that this zooplankton is mainly distributed on the shelf^42^. Indeed, at-sea presence of little auks in NW and central Spitsbergen is restricted to the continental shelf^52^. Depths down to 250 m at the foraging positions of birds from Hornsund and Bjørnøya are therefore concordant with depth range preferred by Arctic zooplankton communities in the Hornsund area (multiyear cut-off point value of bottom depth 242 m^31^). Regular presence of the open and deep-sea copepod, *Calanus hyperboreus* in food samples collected in the ‘high-Arctic’ Magdalenefjorden colony^43^ indicates foraging in deep water beyond the shelf and outside MIZ, i.e. in eddies as confirmed by at-sea surveys^61,62^.

The areas classified by our fuzzy logic model as suitable are generally concordant with the observed at-sea distributions of foraging little auks. The distribution of foraging birds around Hornsund were mainly restricted to the shelf area^12,36,37,63^. Foraging little auks from Magdalenefjorden, on the other hand, were distributed across both the shelf area and MIZ^35,36^. The presence of suitable foraging habitats inside Isfjorden and Kongsfjorden is in concordance with results of at-sea surveys that regularly recorded little auks inside these fjords^52,57^. Both mentioned fjords still contain reservoirs of cold Arctic water in the bottom layers or in basins separated by sills, offering refugia for Arctic zooplankton, and making it possible for *C. glacialis* to survive in the face of climate warming^64^. The small area of suitable foraging habitat around ‘boreo-Arctic’ Bjørnøya is concordant with reported little auk foraging, restricted only to areas northeast of the colony where large Arctic zooplankton is more abundant^14,38^.

### Implications for little auk breeding performance

Comparisons of parental effort and breeding success between two ‘high-Arctic’ colonies (Table 2) indicate that even in the Magdalenefjorden colony where parental effort is high (longer foraging flights, lower availability of preferred food), little auks were able to breed successfully (no negative consequences on chick survival, stress level and body mass of adults and chicks). Moreover, comparison of two contrasting years in Hornsund revealed that in a warm year, adults were able to compensate for a poorer quality and quantity of food delivered to chick by an increase in feeding frequency^30^. However, fledging success in Isfjorden (62–97%), the ‘high-Arctic’ colony characterized by a higher SST in the foraging grounds and without easy access to alternative foraging grounds, was generally lower than in Magdalenefjorden (range 91–100%)^58^. Higher success recorded in the latter colony, if not affected by a lower predation rate (it has not been studied), may have been attributed to possibility of compensation of local suboptimal conditions by foraging in MIZ^65^. Moreover, buffering the suboptimal foraging conditions through behavioural plasticity may have long-term consequences for little auks. An increase in the summer SST resulted in the decrease in survival rates of adult little auks at ‘boreo-Arctic’ and some ‘high-Arctic’ Svalbard colonies, probably through impaired nutritional status during the breeding season^66^. The annual survival rate of adults there was, however, still high (on average 87%), but delayed maturity and poor breeding success implicates that even a small reduction in survival probability can have a strong effect on population dynamics and viability^66^. The southernmost little auk populations from Iceland and S Greenland have already collapsed in numbers following the 19th century shift in sea currents and plankton dispersal^9^. All this indicates that despite the apparent flexibility of foraging little auks, deteriorating oceanographic conditions impose a threat to the population of this endemic Arctic species.

**Table 2:** Comparison of available data on parental efforts and breeding success parameters of little auks breeding in Hornsund (H) and Magdalenefjorden (M).

Variable	Comparison of the colonies	References
Foraging trip distance	H < M	36
Foraging trip duration	H < M	13,43,56
Chick survival	H = M	36,56,65
Body mass of adults	H = M	13
Stress level of adults	H = M	13
	H > M	86
Stress level, body mass of chicks	H = M	13,56,65,86
Chick growth rate	H > M	36,65

### Future habitats

Our results of restricted type response under scenarios of increased SST are alarming. Especially under +2 °C scenario, this response type model predicts considerable losses of suitable habitat within the 130 km buffer zone around all colonies. As expected, ‘boreo-Arctic’ Bjørnøya is the most prone to climate changes according to both response types. Indeed, this area has already been colonized by temperate avian species such as the great skua (*Stercorarius skua*) and northern gannet (*Morus bassanus*)^26^, apparently benefiting from climate warming. Prediction of the flexible response model are more optimistic. This model predicts a stable area of suitable habitat for the majority of the considered colonies (+1 °C scenario) or a moderate habitat loss except for ‘boreo-Arctic’ Bjørnøya experiencing total or massive habitat loss (+2 °C scenario). This is also concordant with prediction that the distribution of the main little auk prey, *C. glacialis*, in the Barents Sea under the +2 °C warming scenario will be similar to present state, i.e. restricted to the shelf zone currently characterized by temperatures 2–4 °C^42^. Thus, temperature increase under +2 °C scenario will not exceed 6–7 °C, recognized as the critical threshold for this copepod functioning.

Foraging behavioural plasticity of the little auk may allow them to adapt to climate change by exploring new foraging habitats (e.g. glacier melt water fronts^67^) and/or feeding on novel zooplankton or even small fish that are extending their distributions in the N Atlantic as a result of global warming. Nevertheless long-term negative consequences for little auks and all Arctic endemic species are inevitable. A continuous warming of the water masses and a new phytoplankton bloom regime in the Arctic Ocean north of Svalbard will cause an ecosystem shift from the current *C. glacialis/C. hyperboreus*-based energy transfer to *C. finmarchicus*-based food chain^68^. While adult little auks might be able to cope with suboptimal prey like *C. finmarchicus* in their environment^69^, the prey selectivity index showed strong preference for breeding birds to provide their chick with large copepods^13,70^. Under such conditions, little auks will be forced to search for preferred Arctic prey among very abundant but energetically suboptimal boreal counterparts.

The retreat of sea ice (some scenarios predict a sea-ice-free Arctic summer by the end of the 21st century^1^) may have a negative effect on several seabirds including those tightly associated with sea ice for their entire annual cycle as the ivory gull (*Pagophila eburnea*) and Mandt’s black guillemot (*Cepphus grylle mandtii*) as well as other species less bound to sea ice, feeding along ice edges, including the little auk, and Brünnich’s guillemot (*Uria lomvia*)^6,8,26^. A northward shift of the sea ice zone beyond the range of little auks will deprive them of foraging in MIZ^35,36^ offering high-energy food. Unlike birds from lower latitudes that can shift their distribution poleward following their prey responding to ocean warming, Arctic seabirds dependent on sea ice have to adapt to changing marine environment^6^, not able to shift northward due to lack of suitable breeding habitats.

### Implications for Arctic wildlife

The little auk is the most abundant seabird in the Atlantic Arctic^9^, and therefore plays a major ecological role within marine ecosystems. Moreover, the presence of little auk colonies profoundly alters terrestrial Arctic ecosystem by providing nutrients^17,71–73^. Such nitrogen-rich ‘oases’ massively enhance primary production resulting in a higher richness of plant cover attracting herbivores including geese, reindeers and invertebrates^16,74^. Hence, the worsening feeding conditions for planktivorous seabirds, such as little auk, may cause them to retreat, potentially inducing serious negative implications for the whole marine and terrestrial Arctic ecosystems.

### Limitations of our models

The limited temporal sample (two years only) may represent a limitation of out habitat modelling. The general pattern of warm and cold water masses is, however, stable regardless of the year as water masses in this part of the Barents Sea follow the bottom topography^22^. We thus consider our results to be representative for the general spatial pattern of environmental conditions experienced by little auks during the chick-rearing period.

The forecast abilities of our models depend on the little auk’s abilities to adapt to rapid environmental changes. Their foraging plasticity, documented here and in other papers^13,30,36,38,43,75,76^, may be population-specific and reflect micro-evolution or phenotypic plasticity of distinct local populations^76^. Under such a local-scale, restricted type response scenario, some local populations (e.g. ‘boreo-Arctic’) could adapt to new conditions, while others, more conservative populations (e.g. ‘high-Arctic’) could become extinct, or leave the current breeding sites seeking for new suitable habitats (if still available).

Real future distribution of little auks may also be affected by other climate change-induced factors, e.g. emergence of new parasites and diseases, increased intra- and inter-specific competition, predation pressure, exacerbated impacts of contaminants and demographic processes^26,77,78^. For example, a combination of heat and mosquitoes, leading to heavy blood-sucking, caused breeding failures and adult mortality in Brünnich’s guillemots breeding in Hudson Bay^79^. In the same species the net effect of combined mosquito parasitism and predation by polar bears reduced overall colony productivity by 20% and increased adult mortality by 20%. Moreover, although warmer waters are expected to be very productive, most of the energy would be transferred to zooplanktivorous fish, such as herring (*Clupea harrengus*), sprat (*Sprattus sprattus*), and mackerel (*Scomber scombrus*), as seen currently in the Norwegian Sea. In consequence, planktivorous seabirds and sea mammals would have to compete harder for food with such small predators and consequently would most likely see reduced abundances, as seen in the boreal regions today^80^.

### Device effects

We based our models on behaviour of GPS equipped individuals. The behaviour and energetics of birds may be directly affected by externally attached devices in two primary ways: (1) by expending extra energy countering both the additional mass and the increased drag; and (2) by decreasing some aspects of their performance, such as speed^81–83^. Studies from Spitsbergen and Greenland revealed that little auks equipped with the same type of GPS-logger as in the present study showed longer trip durations^70^, or performed shorter but more frequent long foraging trips^84^ compared to unburdened individuals. This bias might have affected the pattern of foraging trips observed in the present study. Thus, we postulate to use smaller and lighter loggers in future studies to minimize negative impact on birds performance. Nevertheless, as birds from all studied colonies were equipped with the same type of GPS loggers, possible bias was similar in all studied sites. Moreover, as birds unequipped with GPS-loggers, observed during at sea surveys, were reported from the same foraging grounds as equipped ones in the present study, we consider that our results represent actual characteristics of little auk foraging.

## Conclusions

Our study shows that little auks breeding in Svalbard adopt a flexible foraging strategy, allowing them to forage in a wide range of environmental conditions. As such, they may be temporarily resilient to moderate climate changes of the Arctic marine environment. Nevertheless, our models also predict considerable deterioration of the foraging habitats of little auks in a longer perspective with inevitable negative consequences for this species as well as across whole Arctic marine and terrestrial ecosystems.

## Electronic supplementary material

Supplementary Information
