# Supplementary material for: Habitat foraging niche of a High Arctic zooplanktivorous seabird in a changing environment
Source: Sci Rep. 2017 Nov 24;7:16203. doi: 10.1038/s41598-017-16589-7 (PMC5701252; doi:10.1038/s41598-017-16589-7)
Supplement: Supplementary file 1 — Supplementary Information [file 41598_2017_16589_MOESM1_ESM.pdf]

Supplementary Information

Habitat foraging niche of a High Arctic zooplanktivorous seabird in a changing environment

Dariusz Jakubas, Katarzyna Wojczulanis-Jakubas, Lech M. Iliszko, Hallvard Strøm & Lech Stempniewicz

Supplementary Methods

Fuzzy logic approach

Fuzzy logic is a knowledge-based method, developed to represent imprecise or uncertain knowledge, for describing complex or ill-defined systems. It retains the uncertainty information of each class by taking into account the gradual change from membership to non-membership [1,2]. The fuzzy logic approach is advantageous compared to conventional habitat modelling methods; it allows for the numerical processing of expert qualitative knowledge, and it can consider multivariate effects of variables without the assumption of independence of input parameters. This allows for the inclusion of numerous combinations of factors (summarized in [2]). The fuzzy suitability maps are considered as more informative than conventional suitability maps as they provide extra information about the partial degree of suitability across space. Moreover, fuzzy suitability maps achieve better predictive accuracies than their classic map-overlay approaches (pass/fail screening, graduated screening, weighted linear combination) [3].

Fuzzy models due to their transparency and user friendliness are widely applied in environmental modelling, especially in species distribution modelling [4]. This approach is especially useful in the cases where little systematic field investigations have been conducted. It allows for the use of readily available expertise of specialists in the modelling process to

estimate habitat suitability for a study species. On the other hand, the requirement of expert knowledge, which may be subjective, is considered as the main bottleneck of this approach. Complementing fuzzy systems by data-driven techniques can solve this knowledge acquisition bottleneck [4]. Thus, in the present study we investigated environmental factors affecting Little auk foraging using a simultaneously data-driven technique (conditional inference tree) and fuzzy logic model based on expert knowledge.

In our analyses, we followed the main steps of fuzzy logic systems [3]:

I) Choice of factors (input variables). We chose four factors (input variables) recognized as important for Little auk foraging based on expert knowledge from previous studies (see details below) and results of conditional inference tree analyses assessing the importance of environmental variables (see in the main paper text).

II) Fuzzification (converting the raw environmental measurements into fuzzy membership on the basis of some expert defined fuzzy membership function for each suitability factor). The input data are transformed to a 0 - 1 scale based on the possibility of being a member of a specified set. 0 is assigned to those locations that are definitely not a member of the specified set, 1 is assigned to those values that are definitely a member of the specified set, and the entire range of possibilities between 0 and 1 are assigned to a level of possible membership (the larger the number, the greater the possibility).

We used the following fuzzy membership function:

1) SST in July - fuzzy linear transformation function. This algorithm applies a linear function between the user-specified minimum and maximum values. Area where the slope of the line is decreasing defines the transition zone. We assigned a membership value of 1 (suitable) for any values from -1°C to 6°C and 0 (unsuitable) for any values > 8°C. Range of temperatures between 6°C and 8°C, where the slope of the line is decreasing defines the transition, i.e. suboptimal zone (Supplementary Fig. 1A). Suitable temperature values refer

to 90-95% probability of *C. glacialis* presence in the Barents Sea [5]. The temperature ~6°C reflects the physiological threshold of the species' functioning [6]. Maximal value (i.e. the upper limit of the suboptimal zone) refers to maximal temperatures recorded in sampling points in the Barents Sea with *C. glacialis* [5] and at foraging positions of Little auks around 'boreo-Arctic' Bjørnøya [7].

2) sea depth - fuzzy small transformation function. This algorithm is used when the smaller input values are more likely to be a member of the set. The defined midpoint identifies the crossover point (assigned a membership of 0.5) with values greater than the midpoint having a lower possibility of being a member of the set and values below the midpoint having a higher possibility of membership. We set the midpoint at 242 m. This value refers to the multiyear cut-off point value of the bottom depth for Arctic zooplankton communities in the shelf in the Hornsund area [8]. Thus, values shallower than 242 m have a higher possibility of being optimal for foraging Little auks (Supplementary Fig. 1B).
3) presence of marginal sea ice zone in July (presence scored as 1 and absence as 0) - we employed this factor in our model given that the probability of the presence of *C. glacialis* in the Barents Sea is positively related to sea ice concentration [5], and ice-associated zooplankters serve as regular diet components of Little auks from colonies in cost-effective distances from sea ice [9–11].
4) distance from the colony to the foraging grounds - as foraging is profitable only in cost-effective distances from colonies, in our mode we employed a Cost Distance model based on Euclidean distance from the colonies and estimated costs of flight (1 over sea, 10 over land). We considered flights over land as 'costly' as both observations and GPS-tracks of little auks from Hornsund [12] (Jakubas et al. – unpublished data) indicate that they never flew over land, even if they might have accessed profitable foraging areas using such a route. During fuzzification, we used a fuzzy small transformation function with a midpoint

value 219 km (Supplementary Fig. 1C) referring to maximal foraging ranges of little auks estimated from flight duration [13]. Thus, flights below 219 km have a higher possibility to be undertaken by little auks.

III) Defining a fuzzy inference engine refers to the rules of association to combine the evaluation of multiple factors. We used a weighted linear combination model using a weighted averaging (ANDOR) operator. This operator, in contrast to standard fuzzy intersect (AND) and standard fuzzy union operations (OR), allows for the compensation of a low rating on one factor by a high rating on another factor [3]. We weighted the particular factors based on expert knowledge (Table 1) giving the highest scores to factors important for *C. glacialis*, i.e. SST and presence of the marginal sea ice zone [5]. Particular weights in the weighted linear combinations method fall between 0 and 1 and all the weights for different factors are summed to 1.

IV) Preparation of a fuzzy suitability map providing a continuous suitability field with values ranging from 0 to 1, integrating the information from fuzzificated factors.

V) Defuzzification of the map and preparing a conventional suitability map (defuzzification integrates in one number the information from a fuzzy suitability map, providing two habitat suitability values: 0 and 1, representing unsuitable and suitable habitats, respectively). We determined the defuzzification threshold adapting the method used in calibrating GIS-based predictive models following [3]. By varying the threshold for fuzzy membership from 0.5 to 1.0 with a small increment (0.05) in each step, we compared the actual position of little auk foraging grounds of GPS-tracked birds with predicted distribution of suitable habitat. We then extracted fuzzy membership values and calculated the total number of positions with values above and below the given threshold. We further calculated the positive predictive power for particular thresholds for the whole data set and for "boreo-Arctic" and 'high Arctic' subgroups. Finally, we chose the following two thresholds with the highest positive predictive

power as the optimal defuzzification threshold to predict the distribution of little auk foraging positions:

1) 0.9 - with high accuracy for 'high-Arctic' Spitsbergen colonies (97%) but worse performance for all colonies (64%). This represents a conservative response to climate change with foraging restricted only to optimal, cold water areas reflecting current feeding habitats of populations breeding on Spitsbergen.
2) 0.7 - with a little bit lower accuracy for all colonies (92%) but higher for 'boreo-Arctic' foraging areas around Bjørnøya (87%, in contrast to 41% for 0.9 threshold). This reflects a Svalbard-wide plasticity in little auk reactions to environmental changes, meaning foraging in a wider range of temperatures reflecting the full range of current feeding niches including both 'high-Arctic' and boreo-Arctic' conditions.

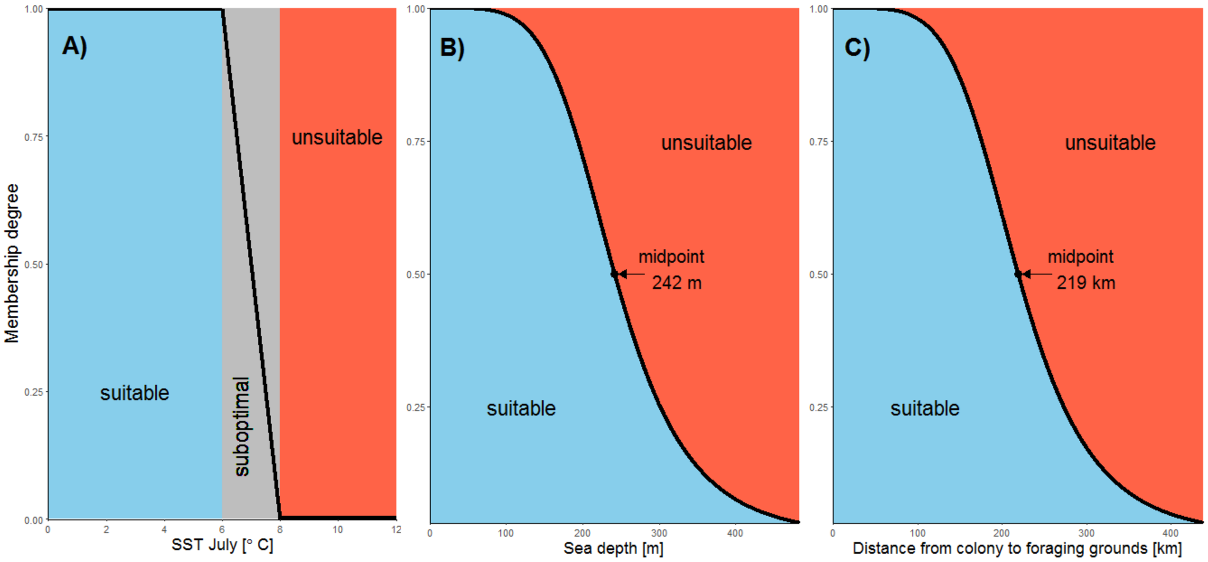

Supplementary Figure 1. Membership functions for the input variables: SST in July (fuzzy linear transformation function; A), sea depth (fuzzy small transformation function; B) and distance from the colony (fuzzy small transformation function; C). Plot was created in R software version 3.3.2[14].

References

1. Zadeh, L. A. Fuzzy sets. *Inf. Control* **8,** 338–353 (1965).

2. Ahmadi-Nedushan, B., St-Hilaire, A., Berube, M., Ouarda, T. & Robichaud, E. Instream flow determination using a multiple input fuzzy-based rule system: A case study. *River Res. Appl.* **24,** 279–292 (2008).

3. Qiu, F., Chastain, B., Zhou, Y., Zhang, C. & Sridharan, H. Modeling land suitability/capability using fuzzy evaluation. *GeoJournal* **79,** 167–182 (2013).

4. Mouton, A. M., Alcaraz-Hernández, J. D., De Baets, B., Goethals, P. L. M. & Martínez-Capel, F. Data-driven fuzzy habitat suitability models for brown trout in Spanish Mediterranean rivers. *Environ. Model. Softw.* **26,** 615–622 (2011).

5. Carstensen, J., Weydmann, A., Olszewska, A. & Kwaśniewski, S. Effects of environmental conditions on the biomass of Calanus spp. in the Nordic Seas. *J. Plankton Res.* **34,** 951–966 (2012).

6. Alcaraz, M., Felipe, J., Grote, U., Arashkevich, E. & Nikishina, A. Life in a warming ocean: Thermal thresholds and metabolic balance of arctic zooplankton. *J. Plankton Res.* **36,** 3–10 (2014).

7. Jakubas, D. *et al.* Foraging behavior of a high-Arctic zooplanktivorous alcid, the little auk, at the southern edge of its breeding range. *J. Exp. Mar. Bio. Ecol.* **475,** 89–99 (2016).

8. Kwasniewski, S. *et al.* Interannual changes in zooplankton on the West Spitsbergen Shelf in relation to hydrography and their consequences for the diet of planktivorous seabirds. *ICES J. Mar. Sci.* **69,** 890–901 (2012).

9. Jakubas, D. *et al.* Intra-seasonal variation in zooplankton availability, chick diet and breeding performance of a high Arctic planktivorous seabird. *Polar Biol.* **39,** 1547–1561 (2016).

10. Jakubas, D. *et al.* Foraging effort does not influence body condition and stress level in little auks. *Mar. Ecol. Prog. Ser.* **432,** 277–290 (2011).

11. Kwasniewski, S. *et al.* The impact of different hydrographic conditions and zooplankton communities on provisioning Little Auks along the West coast of Spitsbergen. *Prog. Oceanogr.* **87,** 72–82 (2010).

12. Jakubas, D. *et al.* Foraging closer to the colony leads to faster growth in little auks. *Mar. Ecol. Prog. Ser.* **489,** 263–278 (2013).

13. Welcker, J. *et al.* Flexibility in the bimodal foraging strategy of a high Arctic alcid, the little auk *Alle alle*. *J. Avian Biol.* **40,** 388–399 (2009).

14. R development Core Team. R: A Language and Environment for Statistical Computing. R Foundation for Statistical Computing, Vienna, Austria. (2015).
